# Research on the Performance of the Spastic Calf Muscle of Young Adults With Cerebral Palsy

**DOI:** 10.4021/jocmr483w

**Published:** 2011-02-12

**Authors:** Renee Lampe, Jurgen Mitternacht

**Affiliations:** a Center for Cerebral Palsy Munchen, Neuro-Orthopaedic department of the Clinic 'Klinikum rechts der Isar' of the Technical University Munchen, Germany; b Orthopaedic department of the Clinic 'Klinikum rechts der Isar' of the Technical University Munchen, Germany

## Abstract

**Background:**

The aim of this study was to find an objective graduation of pes equinus in infantile cerebral palsy, especially with regard to functional aspects, to allow a differentiated choice of the therapeutic options. Very often raises the question of whether a surgical lengthening of the Achilles tendon may let expect a functional improvement.

**Methods:**

For this documentation 17 patients with pes equinus and a diagnosis of spastic cerebral palsy, primarily of the lower limbs, and hemiplegia were examined first clinically and then by a procedure for calculating the functional kinetic parameters from an in-shoe plantar pressure distribution measurement (novel pedar-X system), which is used in many orthopedic practices and clinics as a standard measuring device. Using additional video motion analysis, the flexion in the ankle joint and the ankle joint torque were determined. From this the physical performance of the spastically shortened calf muscle was calculated. The course of the curves of torque and joint performance allows a functional classification of the pes equinus.

**Results:**

Approximately three quarters of all pes equinus demonstrated functional activity of the most part of the normal push-off propulsion power. Even the rigid pes equinus was capable of performing push-off propulsion work, provided it converted energy that was absorbed during the heel-strike phase and released it again during the push-off phase. This suggests that the function of paretic ankle joint is better than its kinematics of motion.

**Conclusions:**

A heel strike with a pes equinus triggers via stretching stimuli in the muscle-ligament structure reflex motor functions, thereby causing the typical spastic gait pattern. This remarkable gait pattern is often evaluated as dysfunctional and as absolutely requiring correction. However, an aspect possibly neglected in this instance is the fact that this gait pattern may be efficient for the patient and may in fact be a suitable means allowing for economic locomotion despite the cerebral control deficits.

**Keywords:**

Pes equinus; Cerebral palsy; Pedography; Ankle joint performance

## Introduction

Infantile cerebral palsy is the result of permanent damage to the brain which occurrs during the brain's most important developmental period, i.e., the time before, during or after birth. The damage usually concerns the area of the first motor neuron and therefore quite frequently the nerves and neuronal pathways of the central nervous system that allow for active movement.

Infantile cerebral palsy is the cause for a defective regulation of the muscle tone resulting in spasticity, dystonia or ataxia. Most frequently, the clinical picture presents with spasticity [1].

The spastic type of cerebral palsy is characterized by an increased muscle tone in the muscles of the extremities. Typically, the lower limbs show deformities like pes equinus, club foot and flat foot. The initially correctable and later fixed pes equinus occur with spastic hemiparesis, diparesis and tetraparesis. Pes equinus is the most common deformity of the lower extremities and the first to be noticed [2, 3]. Its incidence is determined by several factors: On one hand, there is dysfunction of the neuromuscular coordination accompanied by a pathological dysbalance of the muscle tone. This can be caused by persistent pathological reflex reactions and defective voluntary innervation. On the other hand, mechanical factors caused by missing or misapplied loads have to be taken into account. For example, delayed learning of how to walk and frequent sitting aggravate the flexion contractures and contractures of the M. gastrocnemius. Unequal leg lengths will often lead to a pes equinus. The patient thereby compensates for the different lengths of the legs.

In this publication we want to present calculations of the ankle joint moment and the performance of the spastic calf muscle by using in-shoe plantar pressure measurements and synchronous video analysis. Knowledge of the spastically shortened muscle's performance contributes to a basic understanding of the spastic gait pattern and spastic locomotion and is useful when considering surgical procedures.

The patients walk on a treadmill wearing their own shoes. The neuromuscular functions interacting with the movements of the joints only manifest themselves when movement is performed. To continuously deviate the in-shoe pressure distribution, the shoes are equipped with the novel measuring system pedar-X. The device records a "movie" of the pressure distribution in the unrolling of the foot. It takes 100 pressure distribution images per second. The measurement period is about four minutes, corresponding to about 140 step cycles.

By integrating the pressure over all segmental areas, pressure distribution image after pressure distribution image, the temporal progression of the total ground force can be calculated. By integration of the pressure values, considering the lever distance relative to the projected axes of the ankle joint, the external joint moment can be calculated. To this end, the musculature of the lower leg must generate an internal moment that exactly compensates for the external moment.

In conclusion of the investigation, conservative and surgical treatments to improve joint deformities particularly of the lower extremities are considered.

## Materials and Methods

### Criteria for the selection of patients

All patients were aged 10 - 17 years. They were all able to walk without support and were therefore classified as GMFC I or II. Nine patients had spastic hemiparesis and ten had spastic diparesis. As they had been participating in sports therapy, they were already familiar with walking on a treadmill. None of the patients had had any corrective surgery until that time. All patients had been treated orthopedically because of their foot deformities and had been provided with orthopedic insoles to support heel and arch.

The clinical examination focuses on an assessment of joint deformities of the lower extremities and the range of motion of the knee and ankle joints.

### In-shoe plantar pressure distribution measurement

**Figure 1.**
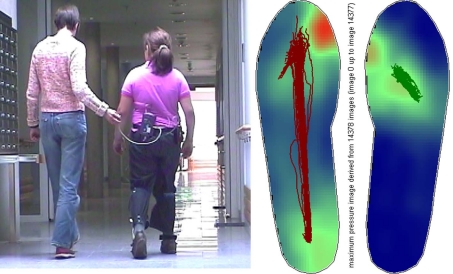
Example of a measurement: Pedogram with gait lines of a female patient with pes equinus on the right side.

In certain ways the sole of the foot represents the physical interface between a person and his or her environment. All forces and the entire body weight are transmitted over the sole to the ground. The foot is the most distant limb of the body. In terms of its functionality, however, the foot occupies a central position in the musculoskeletal system. Therefore, it makes sense to depict the function and magnitude of the forces that occur here by the application of the pedar-X measurement method. The measurement insoles are usually employed especially for fitting orthopedic insoles or, for example, for assessing the effectiveness of an orthopedic shoe on a deformed foot. The measurement insole is laid into the shoe and connected to a data logger that is carried by the patient (Fig. 1). The measurement insoles are available for all shoe sizes and are chosen for this study according to the individual shoe size of the patient.

As the measurement insole does not shift in relation to the foot when walking, one walking sequence can record a large number of paces and the results can be summarized in an averaged pressure distribution figure of an averaged sequence of paces. In order to synchronize the pressure measurement data with the video recording, the data logger at the start of the measurement gives off an LED-flash, which is seen on the video. The measuring rate of the pedar-X system was set to 100 complete pressure measurement images per second. Each measurement insole has 100 individual capacitive sensors that are distributed evenly over the entire area.

The plantar pressure distribution of both feet is then recorded while walking on the treadmill.

### Ground force and torque

Integration of the pressure values across the entire area produces the vertical force at that point in time. The course of the force over time is identical to the progression of the curve of the vertical force component Fz from a ground force measurement. The external moment relative to the axis of the joint is obtained by integration of the pressure values over the entire area while taking into account the lever distance of the center of gravity of the pressure relative to the axis of the talocrural joint (upper ankle joint, UAJ). In accordance with the lever principle, the musculature must counteract this external moment with an internal moment of equal intensity, e.g., the external dorsal moment is counteracted by an internal plantar flexion moment by the tensile force of the triceps. The tibial muscle compensates the external plantar-flexion moment during the heel strike, the triceps compensates for the external dorsal extension moment.

The differentiation of the ankle moment from the pressure distribution requires the use of approximations because only the vertical component of the actual three-dimensional ground force vector is considered and because the axis of the talocrural ankle joint is not inside the measuring plane but several centimeters above it. But a more precise error calculation shows that the error is only a few percentage points at the most.

### Measurements on the treadmill

The measurements for the following analysis of the present study were done on the treadmill (compare to Figure 4). The use of the treadmill allows for establishing a defined and constant walking speed. Patients were allowed to adjust the treadmill's velocity to their preferred speed. The length of the measurement can be selected in any which way without the patient having to change the walking direction. This is, first and foremost, the only way that a sufficiently large number of movement cycles can be measured for the video movement analysis while keeping the complexity at an acceptable level. Most study volunteers also underwent plantar pressure distribution measurements while walking on a flat plane (Fig. 1). This allowed for monitoring as to whether the gait pattern on the treadmill would maintain its natural structure. With regard to the gait parameters of the plantar pressure distribution and the force parameters derived from them, no essential differences were documented for the gait characteristics that are described below. But it was not possible to record any angular curves while walking on a flat plane.

**Figure 2.**
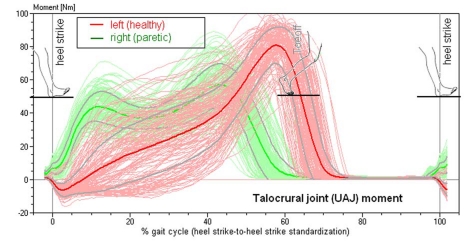
Torque of the talocrural joint in a patient with spastic hemiparesis on the right side (compare to Figure 4). Superimposed 156 individual steps, median curve and standard deviation.

Figure 2 shows the torque curves derived from the plantar pressure distribution during the gait cycles. The torque curves document the lateral asymmetry of hemiparesis with special acuteness. While the curve progresses as described on the healthy side, it changes on the paretic side becoming similar to the curve of the ground force. These documents that the lever arm of the torque remains almost constant, and the point of application of force is in the forefoot from the time of the first placement of the foot until the time of push-off from the walking surface (see pedogram in Figure 1). Also typical is the reduced maximum torque height reflecting the weakness of the spastic musculature.

**Figure 3.**
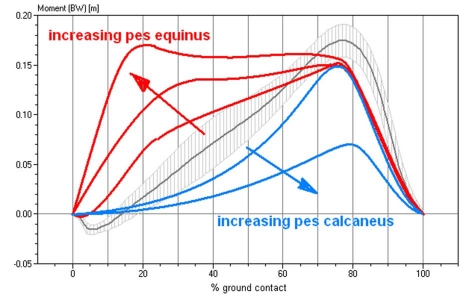
Characteristics of the torque curves of the talocrural joint in different functional pes equinus type, ranging from a completely rigid pes equinus to a flail paralytic pes equinus. This diagram standardizes the torque for the respective body weight. Gray and hatched in the background, the averaged talocrural joint torque in 103 healthy study volunteers.

Figure 3 shows a schematic depiction of the different torque progressions in different manifestations of the pes equinus.

### Analysis of the ankle joint angle motion

**Figure 4.**
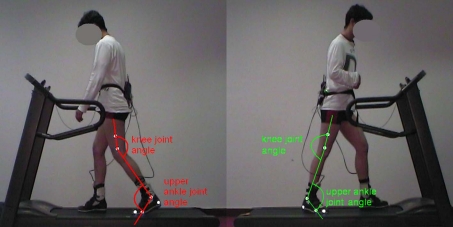
2D movement analysis with reflective markers and joint angle measurement in a patient with spastic hemiparesis on the right side.

The ankle joint angle was measured synchronously to the plantar pressure distribution while walking on the treadmill. The joint angle was determined by the position of light reflecting markers attached to the patient's legs (Fig. 4). A SIMI motion system was used for video analysis. The video analysis system permits digitization of the kinematic parameters of the ankle joint from a distance without touching the patient.

In order to be able to arrive at definite statements as to the characteristic gait pattern of the patient, a sufficient number of gait cycles (between 50 and 100 in this study) need to be recorded.

**Figure 5.**
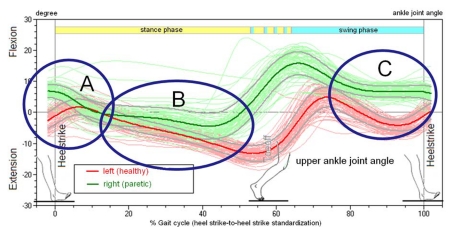
Joint Angle of the talocrural joint (upper ankle joint, UAJ) during the gait cycles, orthopedic notation, patient from Figure 4.

The upper ankle joint (UAJ) angle curves are represented in Figure 5 by way of an example. The curves of the paretic and of the non-affected legs of hemiplegic patients can be compared. In this patient with spastic hemiparesis on the right side, typical differences can be seen between the paretic and the healthy legs. In the heel-strike phase (A) the initially lifted healthy foot folds toward plantar assuming an increased flexed position. The paretic foot makes first-strike contact in the pes equinus position, i.e., in the plantar flexion position. Then the heel bends to plantar, the talocrural joint performs a movement in the direction of a dorsal extension. In the middle stance phase (B) the lower leg inclines increasingly toward the front; on the paretic side with partially fixed pes equinus this occurs to a considerably lesser degree than on the healthy side. During the second half of the swing phase (C), the paretic foot is lifted to a visibly lesser degree than the healthy foot. Different patients demonstrate considerable characteristic variations during all phases, depending on whether the pes equinus is fixed in place, "plastically flexible" or atonic.

### Calculating the performance of the calf muscle

**Figure 6.**
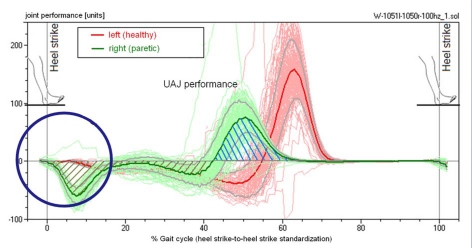
Performance of the calf muscle, patient from Figure 4. The energy that is absorbed during the extension is released again at the end of the contact phase.

Based on the synchronously recorded curves of the angular movement of the talocrural joint (joint angle φ), specifically the angular velocity, which is the temporal derivation of the angular curve, and the torque of the talocrural joint, it is possible to calculate the work output of the talocrural joint (Fig. 6). Integration of the output over a time segment yields work in Joule = Watt-seconds. In Figure 6, the work corresponds to the hatched areas below the curves. Positive areas correspond to work performed by the muscles in a concentric movement released to the outside, i.e., work that is invested into the movement; negative areas are generated with eccentric muscular use, i.e., the muscle extracts work from the movement. On the healthy left side of the study volunteer in Figure 6, the calf muscles generate kinetic energy during the propulsion off the ground. On the paretic side energy is absorbed during the heel strike and converted into straining of the muscle-tendon structure of the talocrural joint and the calf musculature. The energy that is released during the propulsion off the ground corresponds in this case almost exactly to the energy that is absorbed and could be retrieved directly from the mechanical preload, like in an elastic spring, without the muscle generating any new energy in the true sense of the word.

## Results

Based on the characteristics of the angular, torque and performance curves, we will provide a classification of the pes equinus types in the study volunteers.

**Figure 7.**
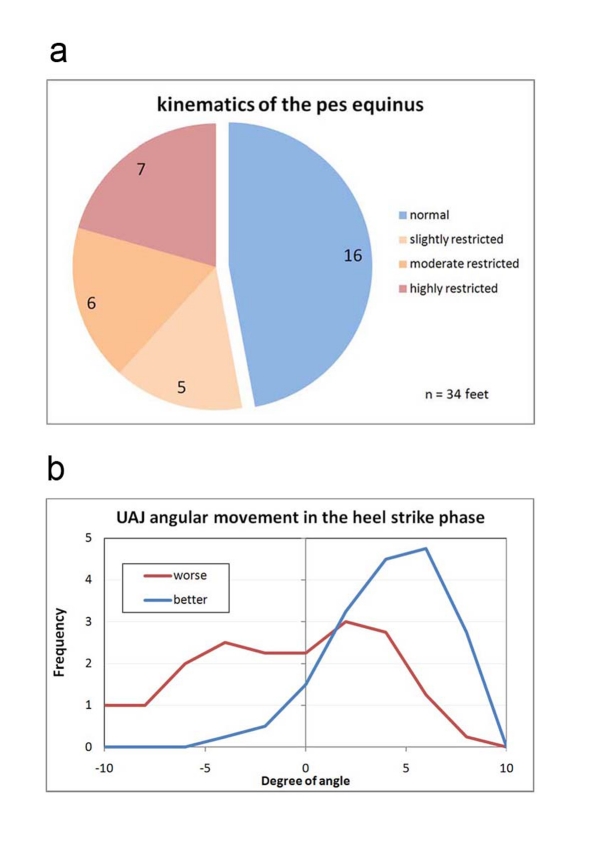
(a) Classification of the angular movement of the talocrural joint in the folding phase during heel strike. Normal: Angle progression during heel strike with lifting and folding as in a healthy person, meaning primarily, in a stride, the foot is placed with the heel first while the forefoot is being actively lifted. Slightly restricted: The forefoot is only minimally lifted during the heel strike event. Moderately restricted: The foot is placed more or less flat. Highly restricted: The foot hangs in plantar flexion during the heel strike event and the first contact with the ground occurs with the forefoot or the toes. (b) Frequency distribution of the measured folding angle in the talocrural joint during the heel strike phase.

The angular curves describe the kinematics of the progression of the movement. Kinematic parameters are the orders of magnitude that the observer perceives "from the outside" during a clinical gait analysis, even if the observer is unable to quantify them. A direct component of the definition of the "kinematic pes equines" is primarily the angular movement of the talocrural joint during the heel-strike phase (marker A in Figure 5). Figure 7a provides a classification of all 34 feet of the 17 study volunteers. Figure 7b demonstrates the frequency distribution of the measured folding angles. If the presentation of the disability is asymmetrical, the measured value of the more paretic leg is assigned to the distribution *worse*, while the less paretic leg is assigned to the distribution *better*. The measured curve of the paretic legs shifts significantly to lower folding angles and/or dorsal extension movements during the heel strike event of the pes equinus.

Described as *healthy* is, as shown in Figure 7a, the angle progression when the heel is placed with a lifted forefoot, and the forefoot is then folded forward in a controlled motion at an angle of at least 3^o^. This is the case in example Figure 5, marker A on the left foot (red curve). The exactly opposite progression of the green curve (right foot) in Figure 5 corresponds to a *strong* pes equinus position. The stepping event starts with the tip of the foot, with the talocrural joint at a 5^o^ angle or stronger flexion position. With the increase of the load acting on the foot, the forefoot is folded in the direction of a dorsal extension and the heel approaches the ground. In an extremely rigid pes equinus the flexion position remains intact and no ground contact by the heel occurs. Evaluated as *minimal* were instances in which the folding direction corresponded to that of a healthy foot but the extent of the movement was small, specifically if it was markedly less on the paretic side as compared to the healthier side. In instances of flat-footed stepping events without or with a very minimally marked folding phase, the evaluation of *medium* was selected.

Two study volunteers are in the class "healthy" in Figure 5, who fall in this group with both feet. This group thus contains 16 feet by 14 study volunteers. In the other classes the number of the feet corresponds to the number of the study volunteers. Approximately half of the feet in Figure 5 fall into the category *healthy*.

**Figure 8.**
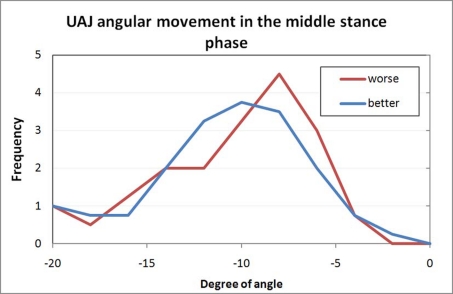
Frequency distribution of the measured folding angle in the talocrural joint during the middle stance phase.

Figure 8 shows the extent of the increasing dorsal extension in the talocrural joint in the middle stance phase. The shift of the curve of the paretic feet to a lesser angular movement is caused by the part of the rigid pes equinus that allows only for minimal dorsal extension. The cluster with very large dorsal extension angles includes the study volunteers with atonic paralysis and pes calcaneus.

**Figure 9.**
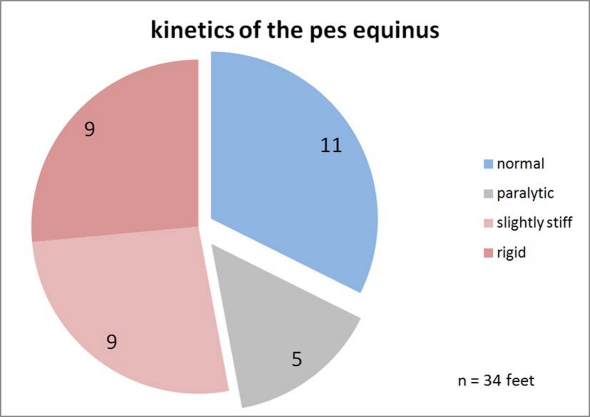
Classification of the upper ankle joints kinetics. Normal: The progression of the torque curve is as in healthy study volunteers. Paralytic: The torque increases with a temporal delay and stays less overall. Slightly stiff: Somewhat premature increase of the torque curve. Rigid: Quick increase of the torque, the curve is similar to the ground force curve because the point of application of force is permanently in the forefoot.

The kinetics of the movement of the talocrural joint is defined by the torque. As described, the torque is calculated based on the order of magnitude of the ground force and the distance of the force vector relative to the axis of the talocrural joint. The classification was defined according to the schematics in Figure 3. The progression of the curve was evaluated as *healthy* if the weight-standardized curve did not deviate by more than two standard deviations from the standard curve and spread of 103 healthy study volunteers. A curve that is at a more delayed incline and/or a curve progression that is too low overall defines the paralytic pes equinus. A prematurely increasing torque characterizes the rigid pes equinus, depending on how pronounced, as *minimally* or *strongly rigid*. Figure 9 provides correspondingly to Figure 7 this classification with regard to the kinetics. Study volunteers are now represented twice in all four classes, respectively one volunteer in the class "healthy" and "rigid" and respectively two volunteers in the class "paralytic" and "minimal". These study volunteers were categorized as having diparesis in the clinical diagnosis. Regarding the aspect kinetics of movement, more feet are shown to be pathological and/or fewer as healthy than in the kinematics.

**Figure 10.**
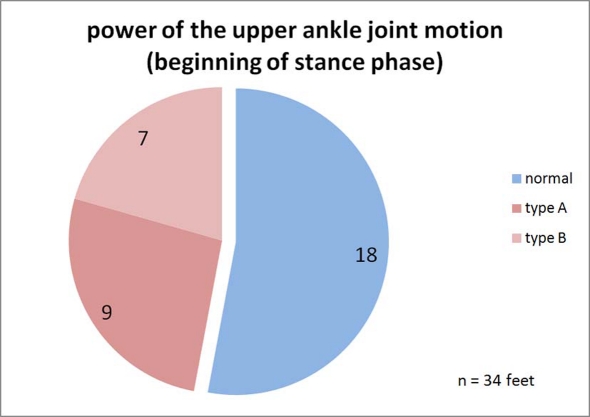
Classification of the power of the upper ankle joint motion. Normal: The performance curve of the talocrural joint in the heel-strike phase is at 0; no movement energy is extracted. Plastic: During the heel-strike phase and beyond the middle of the stance phase, energy is continuously absorbed by the talocrural joint structure. Rigid: Energy is only absorbed during the heel-strike phase and the muscle-ligament structure is loaded like a spring. This energy may be released again during the toe-off phase and would then not be lost.

**Figure 11.**
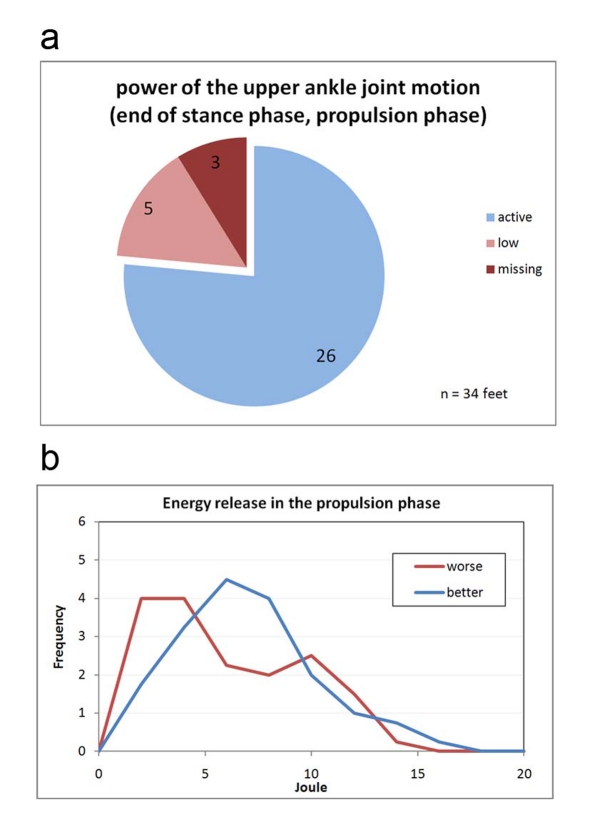
(a) Classification of the power of the upper ankle joint motion. Active: The joint performs work during push-off propulsion. The work can be generated by the calf muscle via metabolic activity or may be recovered in the rigid pes equinus from the spring tension of the heel-strike event. Low: The energy generation can occur as with <Active>; but it is visibly below that of the healthy side (in hemiparesis) and/or that of a healthy study volunteer. Missing: The paretic leg does not generate push-off propulsion energy. In practice, this is only possible in cases of hemiparesis because locomotion is otherwise almost not possible. (b) Frequency distribution of the measured propulsion energies while walking.

The performance capacity of the talocrural joint structure is calculated, as described previously, based on the course of the torque and the angular movement. Figure 10 and Figure 11a show the classification of the progression of the performance curve during the heel-strike and toe-off propulsion phases. During the heel-strike phase, a lack of energy absorption is considered *healthy*, as in a healthy person. The rigid pes equinus, on the other hand, absorbs energy during this phase, as shown in example Figure 6 for the right paretic leg (type A). The third category *plastic* includes pes equinus that, while also absorbing energy during the heel-strike phase, give way during the middle stance phase in the direction of the dorsal extension "*plastically deformable*", continually absorbing further energy during this stage (type B). The number of the feet appearing as healthy is now even larger than previously with the kinetics of the movement. This is because, during the heel-strike phase and the beginning of the middle stance phase, both the paralytic as well as the healthy talocrural joints do not produce any resistance against the dorsal extension movement which is why the performance curve in this phase stays at 0.

Figure 11a classifies the performance and work by the talocrural joint during toe-off. Three feet in Figure 11a are paralytic, meaning they are virtually unable to perform any push-off work at all. These three feet belong to hemiparetic patients who must use the healthy leg alone in order to generate propulsion forces. A patient having two paralytic legs such as this would have a very difficult time at locomotion *per se*. Figure 11b shows the frequency distribution of the measured push-off energies. The curves for the paretic and the healthy and/or healthier legs differ only slightly. The paralytic pes equinus create a slight cluster with very minimal push-off energy. The distributions in Figure 10 and Figure 11a as well as the two curves in Figure 11b demonstrate that the function of the paretic talocrural joint is superior to what the kinematics of movement would give the researcher reason to expect.

## Discussion

The most frequent and most typical comorbidity in infantile cerebral palsy is the spastic pes equinus in its various manifestations [4]. In contrast to the gait cycle of a healthy study volunteer, remarkable changed kinematics are observed most often. The kinematics of the movement is the specific aspect that makes the disability visible to the outside observer.

A heel strike with a pes equinus triggers via stretching stimuli in the muscle-ligament structure reflex motor functions, thereby causing the typical spastic gait pattern. This remarkable gait pattern is often evaluated as dysfunctional and as absolutely requiring correction. However, an aspect possibly neglected in this instance is the fact that this gait pattern may be efficient for the patient and may in fact be a suitable means allowing for economic locomotion despite the cerebral control deficits.

In one study, Lampe [5] determined the muscle volumes of the lower extremities based on MRI tomography images of the lower extremities in adolescents with infantile cerebral palsy and spastic hemiparesis. The examination sought to determine as to what extent muscles atrophy occurs under spasticity and muscles lose volume. Feldkamp [1] describes that spasticity is not equivalent to strength energy. The ability of the spastic muscle to contact is proportionately worse depending on the severity of the spasticity. Lampe [5] believes the reason for the loss of volume is also due to the restricted shortening ability of the hypertonic muscle and assumes a correlation between spasticity and loss of volume of the muscle. Upon reviewing the muscle volumes of different neurogenic foot deformities, significant differences of volume reduction were seen among the individual muscles of the lower leg. The musculature of the lower leg was significantly more reduced than the musculature of the upper leg, respectively in comparison with the healthy side. But the study was not able to demonstrate the actual output power of the paretic musculature.

The goal of the present examination was a documentation of the work output of the paretic muscle during the gait cycle. To this end, measurements of the plantar pressure distribution inside the shoe were recorded during the gait cycle. Beyond the local distribution of pressure, that yields information regarding dysfunctions such as foot drop, flat foot or club foot, it is also possible to quantify kinetic (force) parameters based on the pressure distribution [6]. The resistance moment of the upper talocrural joint is of central significance in the spastic pes equinus. Using light-reflecting markers, the sagittal joint angle movements in the knee and talocrural joint were also recorded. In this way, a determination of the work performance inside the talocrural joint using the gait cycle is also possible.

Approximately three quarters of all feet and/or talocrural joints demonstrate functional activity of the most part of the normal push-off propulsion power. The work output can be generated in different ways; the normal way would be the generation of energy via contraction of muscle fibers. But even the rigid pes equinus is capable of performing push-off propulsion work, provided it converted energy that was absorbed during the heel-strike phase into inner "spring tension" storing said energy temporarily and released it again during the push-off phase. This means the function of paretic talocrural joints is better than their kinematics of movement would suggest. The measured kinetic resistance moments that were also at the forefront during a manual examination also demonstrated stronger changes than the push-off propulsion function of the talocrural joint during the gait cycle.

If the functional output capability of the talocrural joint movement is known, it is possible to draw, based thereon, conclusions as to the necessary treatment, possibly surgery.

The question as to whether a pes equinus in and of itself is to be classified as pathology remains open for discussion. What is uncontested with regard to spasticity though is the fact that any permanent forefoot strain represents a risk for the foot *per se*. The pes equinus position is unstable with regard to the longitudinal axis of the foot. The foot often gives way to a pronated position that can result in decompensation in the Chopart's joint with development of a neurogenic flat foot. This abductus position is often combined with a painful in-toe.

In view of these results, any surgical extension of the Achilles tendon or of the calf muscle must be assessed critically. There may be a risk that the patient's ambulatory capabilities may worsen unexpectedly even though the basic position of the talocrural joint has been corrected to a normal basic position. Moreover, Doderlein [7] demands a careful analysis of any possible functional effects before surgical treatment of foot deformities in patients with spastic cerebral palsy. This must also be taken into account of any assessment of post-surgical results. Dynamic measuring procedures can objectify any functional effects. Senst [8] points to the fact that, contrary to cases involving congenital pes equinus varus, cases of neuroorthopedic pes equinus varus may, due to a shift of the muscular disequilibrium, post-surgically result more likely in an over- or under-correction.
